# *Cladophialophora Bantiana* : A Rare Intracerebral Fungal Abscess—Case Series and Review of Literature

**DOI:** 10.1055/s-0037-1598248

**Published:** 2017-03-30

**Authors:** Maleeha Ahmad, Darren Jacobs, Hueizhi Hope Wu, Donna M. Wolk, Syed A. Jaffar Kazmi, Carlos Jaramillo, Steven A. Toms

**Affiliations:** 1 Department of Neurosurgery, Geisinger Health System, Danville, Pennsylvania; 2 Department of Pathology, Geisinger Health System, Danville, Pennsylvania; 3 Department of Laboratory Medicine, Geisinger Health System, Danville, Pennsylvania; 4 Department of Infectious Disease, Geisinger Health System, Danville, Pennsylvania; 5 Department of Neurosurgery, Lifespan Health System, Rhode Island

**Keywords:** *Cladophialophora bantiana*, pheohyphomycosis, voriconazole

## Abstract

**Background** Intracerebral *Cladophialophora bantiana* may carry up to a 70% mortality rate despite advances in surgical resection capabilities and the use of both systemic and intrathecal antifungal treatments.

**Objectives** The authors examined a retrospective case series of two patients with intracerebral infection from the rare, neurotropic fungus *Cladophialophora bantiana* and conducted a literature review to evaluate optimal therapies.

**Patients/Methods** At our institution, the patients' cases presented with raised intracranial features of headache, visual field cut, and/or memory loss, with a correspondingly wide variety of radiological differential diagnoses. It was the microbiological, histopathological, and genomic identification of *C. bantiana* that ensured targeted, individualized patient therapies.

**Results and Conclusions** Successful treatment depends on obtaining a complete surgical resection, an accurate microbiological diagnoses for mold identification, and an effective long-term, personalized antifungal treatment. Close radiographic surveillance is necessary to ensure complete eradication of pheoid fungi.

With the exception of *Cryptococcus neoformans* , fungal central nervous system (CNS) infections are relatively rare, particularly in immunocompetent hosts. Most fungi live in soil or on vegetation and infect humans only occasionally by inhalation or through puncture wounds. Fungal infections in CNS are almost invariably due to the spread (usually hematogenous) from a primary focus of infection elsewhere in the body. A small proportion of infections complicates direct extension of infections from the air, sinuses, or bone. 1 Yet several fungal species can cause infrequent but often devastating CNS infections due to their recognized neurotropism. These fungal species include *Cladophialophora bantiana* among other lesser known fungi such as *Exophiala dermatitidis* , *Rhinocladiella mackenzie* , and *Ochroconis gallopava* . 2 Despite antifungal therapy and surgical excision, these infections can carry a high mortality of up to 70%. 3

*Cladophialophora bantiana* has been recognized as the most common cause of cerebral pheohyphomycosis. 4 Pheoid fungi share the common phenotypic trait of being darkly colored. This is the result of the presence of dihydronaphthalene melamine in their cell walls. 5 Some have postulated that the melanin pigment contributes to its neurotropism by binding to specific receptors in the blood–brain barrier, allowing it to access the brain parenchyma. 6 Other names given to the agents that cause pheohyphomycosis include dematiaceous or melanized fungi, but the International Society for Human and Animal Mycosis (ISHAM) has recommended the term “phaeohyphomycosis” for any infection that involves a dematiaceous fungus. 7 *Cladophialophora bantiana* itself has different taxonomical names as seen in past literature, including *Cladosporium bantianum* , *Cladosporium trichoides* , *Xylohypha bantiana,* and *Xylohypha emmonsii* . 4 8

Pheoid fungi in general are difficult to identify to the subspecies level. In comparison, *C. bantiana* is relatively easier to identify because of its typical morphology and source of the clinical specimen. It grows on routine fungal culture media. The more commonly used media are potato dextrose agar, oatmeal agar, and malt agar. 2 7 While intrafungal cerebral abscesses have been historically associated with immunocompromised patients, *C bantiana* has a predilection for immunocompetent hosts. 4

The clinical presentation of patients with cerebral pheohyphomycosis may vary but most often presents with features of raised intracranial pressure: headache and seizures. 6 Radiologically, the intrafungal cerebral abscesses are known as “the great mimickers” and may be difficult to distinguish from high grade gliomas, lymphoma, or metastatic cancer. 9 Although the route of infection is unknown, it has been postulated that hematogenous dissemination is the primary mechanism for cerebral pheohyphomycosis. 4 Thus, *C. bantiana* most often results in supratentorial growth with a majority of reported cases growing in the frontal lobes. Contrast-enhanced images show an irregular heterogeneous lesion with mass effect and significant surrounding vasogenic edema.

Hence, successful treatment depends on accurate microbiological and histopathological information for identification of the mold and initiation of personalized, targeted antifungal treatment. New-generation triazole antifungal treatments have been associated with improved survival rates and offer greater benefits than the more traditional antifungal agents once complete surgical excision has been achieved.

## Methods and Subjects

### Case Report: Case 1

**Fig. 1:**
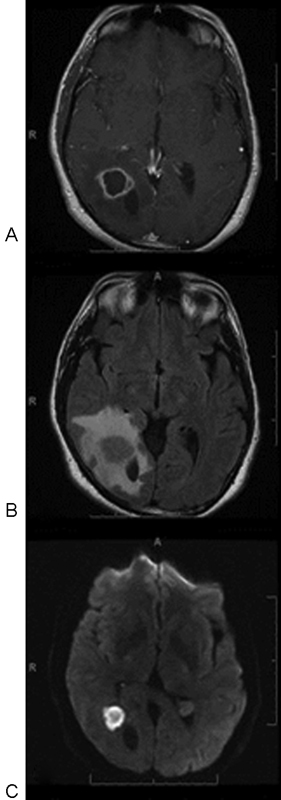
(A) Axial T1-weighted postgadolinium magnetic resonance imaging showing an enhancing cystic lesion adjacent to the right occipital horn of the lateral ventricle. ( **B** ) Large amount of edema on FLAIR (fluid-attenuated inversion recovery) imaging. ( **C** ) Diffusion-weighted imaging (DWI): restriction of diffusion on DWI sequences.

Patient 1 is a 44- year-old male who presented with a week's history of headaches and a subjective left visual field loss, which he noted had occurred while driving and missing an exit that he uses regularly. The patient denied fever or chills. Physical examination demonstrated a left hemianopia. An magnetic resonance imaging (MRI) showed an enhancing cystic lesion adjacent to the right occipital horn of the lateral ventricle ( Fig. 1A ) with a significant amount of edema on FLAIR (fluid-attenuated inversion recovery) imaging ( Fig. 1B ) and restricted on diffusion-weighted imaging ( Fig. 1C ) sequences.

The differential diagnoses for the lesion, including that potentially of intrinsic high-grade glioma, metastases, or an abscess, were discussed with the family who agreed upon proceeding with a surgical resection.

**Fig. 2:**
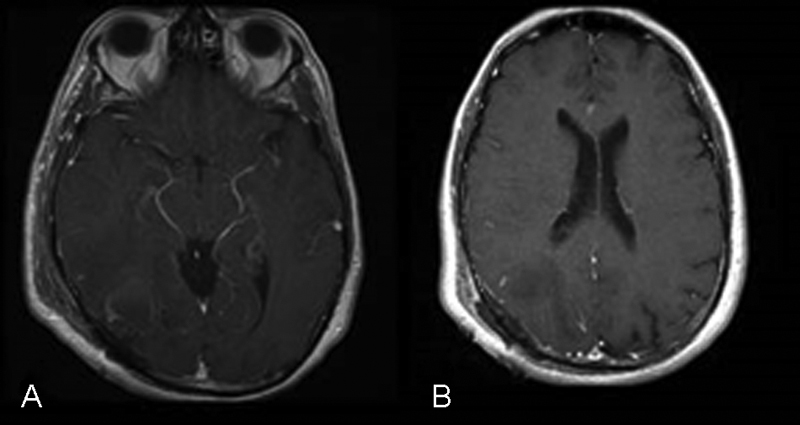
( **A,B** ) Axial T1-weighted postgadolinium magnetic resonance imaging showing lesion resection immediately postoperatively.

Intraoperatively, there was noted to be a clear boundary between the surrounding brain parenchyma, and visibly darker, melanotic, and moderately neoangiogenic lesion. An intraoperative frozen section indicated the possibility of glioblastoma multiforme. Complete resection of enhancing lesion was undertaken including removal of the subependymal lesion entering the ventricle ( Fig. 2A, B ).

**Fig. 3:**
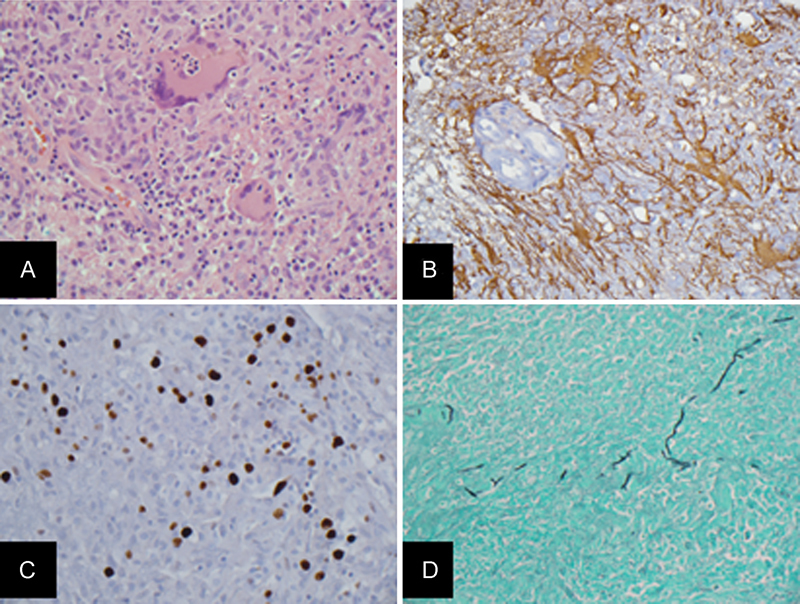
( **A** ) Hematoxylin and eosin stained permanent section demonstrates multinucleated giant cells composed of fungi. ( **B** ) Glial fibrillary acidic protein immunostain demonstrates positivity in reactive astrocytes with mimic to astrocytic tumor cells. ( **C** ) Molecular immunology Borstel-1 (MIB-1)/K _i_ -67 proliferation marker immunostain demonstrates high labeling index with mimic to neoplasm. ( **D** ) Gomori methenamine silver stain highlights fungi.

A series of hyphae were observed on the histopathology permanent sections ( Fig. 3A–D ). The final diagnosis was that of a fungal cerebral abscess, and the patient was placed on intravenous (IV) amphotericin B. Retrospectively, formalin-fixed paraffin-embedded (FFPE) tissue was submitted to the Molecular Diagnostics Section, Clinical Microbiology Laboratory, University of Washington Medical Center (Seattle, Washington, United States), and sequence information was obtained from each of the three fungal targets sequenced (28S, ITS1, and ITS2 regions) with sequence matches to *C. bantiana* . 10 Molecular sequence data were submitted to an open access data repository, GenBank. 11 12 Sequences were annotated and following GenBank accession numbers were assigned: Case1__28S,BankIt1920665 SEq. 1, #KX270255; Case1__ITS1 BankIt1920665, SEq. 2KX270256; Case1__ITS2, BankIt1920665 SEq. 3, KX270257; Case2_ITS1. BankIt1920665 SEq. 4, KX270258. 11 12

The patient then developed the clinical sequelae of fungal ventriculitis and underwent a series of endoscopic debridements. In addition, the patient was commenced on both systemic and intrathecal intraventricular antifungal therapy, including voriconazole. Despite optimal treatment, the patient showed progressive clinical and radiological deterioration and, unfortunately, passed away within 3 months after presentation.

**Fig. 4:**
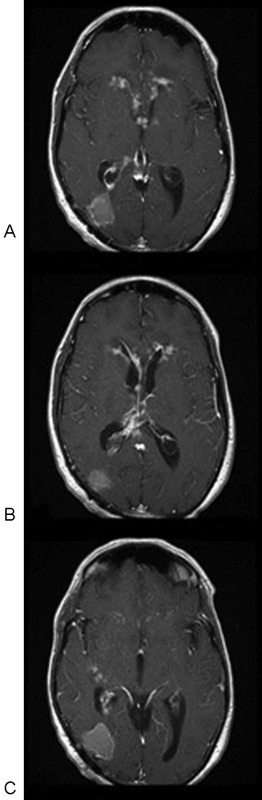
( **A–C** ) Axial T1-weighted postgadolinium magnetic resonance images 1 month postoperatively, showing disease progression in resection bed and diffuse ventricular ependymal involvement.

**Fig. 5:**
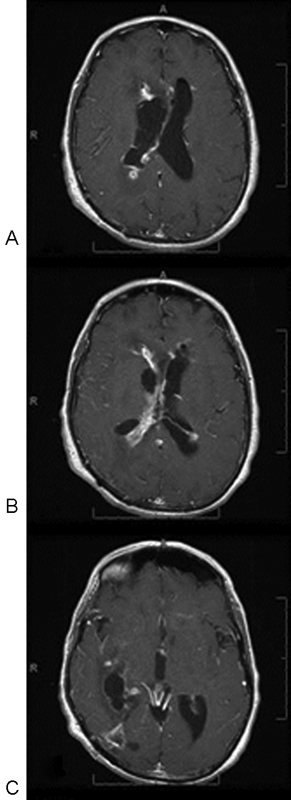
( **A-C** ) Axial T1-weighted postgadolinium magnetic resonance images 2 months postoperatively, showing further disease progression in resection bed and further ventricular ependymal involvement despite endoscopic intraventricular debridements and intraventricular antifungal therapy.

Figs. 4A – C and 5A – C further depict the patient's disease progression and represent a selected series of subsequent MRIs, obtained after his diagnosis and initial surgery from approximately 1 month and 2 months, respectively.

### Case Report: Case 2

**Fig. 6:**
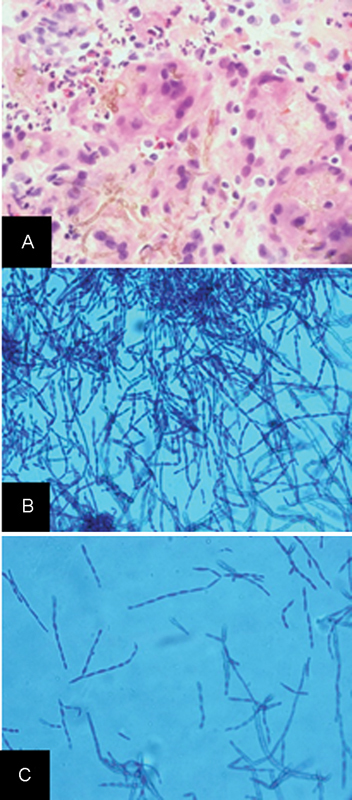
( **A** ) Hematoxylin and eosin stained slide demonstrates multinucleated giant cells composed of fungi. ( **B,C** ) Low (10 × ) and high power (40 × ) magnification of lactophenol cotton blue stains, which show oval conidia in long, wavy, sparsely branched chains, typical of *Cladophialophora bantiana* (Geisinger Medical Laboratories, Danville, Pennsylvania).

The second patient is a 54-year-old male who presented in 2011 with a several weeks' history of a gradual change in mental status with memory loss and headaches. Based on the radiological features and uncertain diagnosis, this patient initially had a biopsy of his right frontal lesion. The frozen tissue section showed pleomorphic cells and branching hyphae. The permanent section with hematoxylin and eosin stained slide demonstrated multinucleated giant cells composed of fungi ( Fig. 6A ). The tissue sample was minced, not ground, and inoculated to Sabouraud Dextrose with Brain Heart Infusion Agar, inhibitory mold agar, and brain heart infusion agar with 5% sheep red blood cells (Remel, Lenexa, Kansas, Untied States); a mold resembling *C. bantiana* was cultivated, and lactophenol cotton blue stains are depicted in Fig. 6B and 6C and show oval conidia in long, wavy, sparsely branched chains. Retrospectively, FFPE tissue was submitted to the Molecular Diagnostics Section, Clinical Microbiology Laboratory, University of Washington Medical Center. In this case, sequence information was obtained from only the ITS1 region with a sequence match to *C. bantiana* . 10

The patient treatment began on IV voriconazole for 2 months. Despite appropriate antifungal therapy, radiological surveillance of this fungal lesion continued to show an increase in size with mass effect. Upon further discussion and agreement of the patient's family, a surgical excision was undertaken. Using neuronavigation, a frontal craniotomy was fashioned, and intraoperatively ultrasound was used for real-time feedback for elucidation and confirmation of resection margins. Meticulous attention was paid to not violate the ependyma in particular, where the lesion encircled the frontal horn at the right lateral ventricle. Microbiology sampling from the second operation was not able to cultivate any fungus on culture growth despite rare hyphae identified on pathological analysis.

**Fig. 7:**
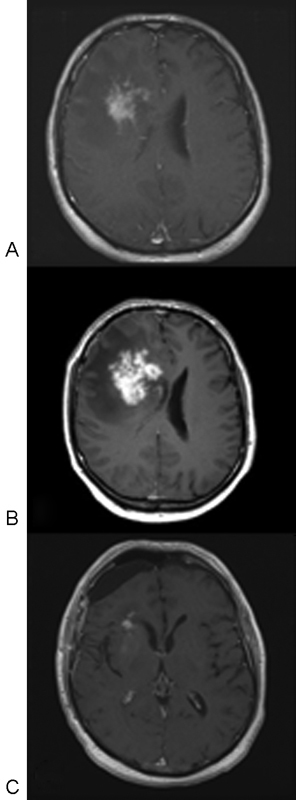
( **A** ) Axial T1-weighted magnetic resonance image (MRI) with gadolinium showing the initial imaging on presentation. ( **B** ) Axial T1-weighted MRI with gadolinium showing increasing mass effect of the right frontal enhancing lesion while on antifungal treatment. ( **C** ) Axial T1-weighted MRI with gadolinium showing good postoperative resection with minimal enhancement at the pole of the right lateral ventricle.

The patient was maintained on long-term IV voriconazole for 6 months with conversion to oral voriconazole for 6 months. MRI scans performed at 6, 12, and 18 months after discontinuation of voriconazole show only minimal residual enhancement ( Fig. 7A–C ), and the patient has recovered well with no intracerebral fungal recurrences.

Both case studies were approved by the Geisinger Institutional Review Board (#2014–061).

## Results

Two patients with intracerebral *C. bantiana* were presented and reviewed. Both cases were successfully cultured and treated with one cured from his pheohyphomycosis. Patients infected with *C. bantiana* usually are male and immunocompetent, and present in their second and third decades of life. 13 There has been no documented predilection for race. In reviews of all reported pheohyphomycoses, 48% have been caused by *C. bantiana* , with a 71% mortality rate. 14 In those patients with culture-proven intracerebral fungal infections, an overall mortality rate of 73% has been documented with no differences in mortality seen between the immunocompromised and immunocompetent patients. 4

Once there has been CNS infiltration of the *C. bantiana* abscess, the symptomatology is one of an intracerebral abscess. Patients most commonly present with a persistent severe headache but less commonly show overt signs of fever, meningitis, or seizures. Imaging often reveals a predilection for the frontal lobe, although reports have shown a variety of lobar and hematologic spread of brain metastasis from cancer, similar to infratentorial sites of involvement.

## Discussion

It is imperative to avoid delay in treatment and to obtain a tissue sample of the lesion as soon as possible. The intraoperative aim should be twofold: first, for microbiological purposes (culture and nucleic acid sequencing), and, second, for maximal resection of the lesion. Intraoperatively, depending on the chronicity of the abscess, the wall of the abscess can also be resected after aspiration. Care needs to be undertaken intraoperatively regarding microscopic fungal residue during handling of surgical instruments. It needs to be noted by the neurosurgical team that despite gross total resection of the fungal lesion, there may have been infiltration by the fungus into the normal appearing brain tissue. Hence, it is essential to undertake close postoperative radiological surveillance. The authors of this paper would suggest both a preoperative and an early postoperative MRI (with gadolinium and FLAIR sequences specifically) ideally within the first 24 hours. At the start of the antifungal treatment, a minimum of two MRI scans should be undertaken in the first 2 months and more frequently if clinical requirement dictates.

Histologically, the abscess cavity contains necrotic material and vascular granulation tissue. Within the inflammatory infiltrate, the fungus can be identified on wet mount preparation showing branching, septate hyphae. From a microbiological perspective, there is no single stain or laboratory test that specifically identifies this fungus. *Cladophialophora bantiana* is noted to grow at 42°C, differentiating it from other saprophytic fungi. 14 Additionally, this fungus does not display pigmented scarring on the elliptical conidia.

A hypothesis is that the melanin within the fungus shows affinity to receptors on the blood–brain barrier, allowing the fungus to penetrate into the parenchyma. 4 This is the same hypothesis that is used for malignant melanoma metastases to the brain. However, fungal melanin is derived from acetate, and human melanin is derived from tyrosine, both differing biochemically but with similar physiochemical properties.

Traditionally, a combination antifungal therapy has been used with amphotericin B, 5 flucytosine (5FC), and an azole. Voriconazole has good CNS penetration and consistent potent activity and is available in IV form. Hence, patients require the need for a long-term indwelling IV catheter. Amphotericin B and 5FC are now less commonly used due to the associated toxicity and modest efficacy against these fungi.

What remains a cause of debate is the source of these primary CNS fungal infections. In published cases, the patients did not have a previous fungal infective focus; hence, local extension is not a possibility. As most published reports of *C. bantiana* infection are of young, immunocompetent males, 13 the possibility remains that illicit drug use caused blood contamination with the fungus that spread the fungus hematogenously. In the two cases presented here, drug use was not a likely source.

*Cladophialophora bantiana* has been reported worldwide and is distributed extensively in nature within brick walls and in soil. The respiratory tract has been postulated as the portal of entry for *C. bantiana* infections. 15 Both cases underwent extensive environmental evaluation to identify a potential source for the infection. The mold was discovered in the walls of the basement of the residence of case 1. Soil samples were positive in case 2, and there had been a recent renovation of the house with reports of removal of mold in wall siding, perhaps contributing to the infection. The virulence factor of *C. bantiana* is also unknown. Recently, this fungus has been added to the Biosafety Level 2 containment list in view of the high mortality seen in patients infected with this intracerebral fungus. 16

The precise ecological niche of the fungus is unknown, but *C. bantiana* is generally believed to be a soil fungus. 14 17 It has been found in geographical regions as diverse as the United States, Japan, India, Belgium, France, South Africa, Mexico, and Brazil, but has rarely been isolated from sources other than clinical samples. 18 *Cadophialophora bantiana* is distributed worldwide, but infections with this organism are especially encountered in subtropical and humid climate areas. 19 In one study where 37 strains of *C. bantiana* were analyzed for antifungal susceptibilities, only one, found in saw dust, was isolated from the environment. 9

*Cadophialophora bantiana* CNS infections are considered a therapeutic challenge, with mortality as high as 70% even with appropriate treatment. 2 18 20 To complicate things further, treatment is largely based on anecdotal experience and expert recommendation. Additionally, due to the paucity of cases, randomized controlled cases are not possible. Many have advocated for combination therapy, 2 17 21 22 which in many cases is complicated by side effects from antifungals. Even though susceptibility testing has not been standardized, antifungal susceptibility testing has been advocated to guide therapy. Our isolate had minimal inhibitory concentration (MICs) to amphotericin B of 0.5 μg/mL, caspofungin 4.0 μg/mL, fluconazole 16 μg/mL, itraconazole 0.06 μg/mL, posaconazole < 0.03 μg/mL, and voriconazole 0.06 μg/mL.

Unfortunately, very few data concerning the correlation between the MIC and the outcome of treatment with amphotericin B are available for dematiaceous fungi. Generally, filamentous fungi are not susceptible to fluconazole with most MICs >16 μg/ml. 21 Most reviewed articles recommended combination treatment with amphotericin plus either 5FC or an azole, either itraconazole or voriconazole. 21 22 In vitro data have suggested that the MICs to the newer azoles such as voriconazole and posaconazole are lower than for amphotericin B, 21 23 24 as was the case for the isolates recovered from our two patients.

The role of surgical evacuation versus antifungal therapy alone is unknown, but evidence is accumulating in favor of complete surgical excision, with most reports of successful therapy involving surgical resection, whereas most reports of treatment failure occurred when surgery was either not done or not possible. 25 26 Complete excision of brain abscesses may lead to better outcomes than simple aspiration or partial excision given the modest response to antifungal therapy associated with these infections. 21

## Conclusion

The neurotropism of fungal infections results in the unusually rapid hematogenous spread of these organisms to the CNS. Intracerebral fungal infections continue to have one of the highest rates of mortality in clinical medicine despite advances in antifungal systemic and intrathecal therapies. We have presented our rare clinical case series of *C. bantiana* infected patients, both of whom underwent maximal surgical resection and microbiological therapies based on pathology and presumptive fungal culture. Two very stark outcomes occurred, thereby illustrating the very grim nature of this disease and the need for rapid methods for fungal genomic identification and deoxyribonucleic acid sequencing, and the early recognition of this mold in fungal culture. Collaboration and communication between neurosurgery, microbiology, and pathology are crucial for rapid recognition of this pathogen. The authors of this paper, thus, advocate a maximally aggressive surgical resection with maximum care taken to avoid cerebrospinal fluid fungal inoculation, followed by adjuvant long-term antifungal therapy and close radiological surveillance.
